# Treatment and Remote Results of Injuries of the Nerves in War Cases

**Published:** 1919-01

**Authors:** 


**Treatment and Remote Results of Injuries of the Nerves in War Cases.** Twenty-seventh French Congress of Surgery, *Presse Médicale*, October 17, 1918.

Mr. H. Delagenière (*from Le Mans*) reports the results obtained by him in treating surgically 358 cases of injuries of nerves. In 236 of these, resection and suture were resorted to; in 113, simple surgical liberation of the nerve proved effective; and in 9 cases the resection was followed by the grafting of the nerve. To these results the author adds 17 cases of " causalgia " treated surgically by sectioning the nerve above the lesion and suturing immediately.

Resection followed by suture is the treatment of choice, giving as high as 88 per cent. of excellent results when done under favorable conditions; but the removal of the injured part of the nerve must be complete, and the suture done between sound ends of the nerve. If the resection extends so far that no direct suture is possible, even with the limb flexed, the operation should be performed in two steps. After two interventions, at an interval of 3 or 4 months, the resection of all the injured region will generally be complete.

The grafting of the nerve is resorted to when the loss of substance is considerable. The graft may consist either of a fragment of nerve removed from an amputated limb (homograft), or of two joined fragments of the musculo-cutaneous nerve. Out of 9 cases of grafting, 3 had good results, and 6 had no results at all.

The liberation of the nerve has good results only in cases of simple compression. If the nerve is injured, the best method is to resort to resection and suturing.

Prof. Delagenière resorted to surgical treatment in some cases of causalgia. The nerve is sectioned just above the lesion and sutured immediately. The neuritis heals while the repairing process of the nerve is going on.

The suture should done be as early as possible, although complete healing was obtained in one case operated 28 months after receipt of the wound.

True motor nerves, such as the radial, musculo-cutaneous, and external popliteal sciatic, repair better and more rapidly than compound nerves.

The repairing process is very long. It can easily be followed by the distant tingling sign on percussion.

The a thor concludes by advising immediate nerve suture, after careful examination of the bruised parts. If primary suture cannot be done, the resection of the nerve should not be attempted until the wound is completely healed. A physiological section in a nerve can generally be diagnosed with certainty after 4 months' time.

Mr. Delorme (*from Paris*) discusses the difficulties that are met with in secondary sutures of injured nerves. In this stage the injured part of the nerve is surrounded by a sort of fibrous sheath. This is due, according to some authors, to a repairing process in the surrounding soft parts, and, according to others, to a neurologic proliferation of the nerve itself. The presence of axis-cylinders in this sheath has led surgeons to respect it during recent years, and to practise simply eccentric liberations and excisions when this sheath seemed responsible for some constriction of the nerve.

Mr. Delorme does not approve of this technic. He considers that the suture of the nerve must necessarily be done between fresh and sound ends. Whether these ends be frankly separated, or joined by the fibrous sheath, it is absolutely necessary to approximate two freshened ends of the nerve.

The neuromae developing on the injured extremities of a sectioned nerve hinder considerably the regular progression of the regenerated axis-cylinders. They must, therefore, be excised deliberately. It is a necessary condition for a successful intervention.

This technic sometimes requires numerous resections. In such cases, Mr. Delorme realizes the approximation of both ends, without tension, by freeing the nerve on a certain length, and placing the limb in such a position as to obtain sufficient relaxation of the nerve.

Even while freed and isolated in that way, the nerve continues to receive the necessary blood supply by means of its small " intranervous " blood vessels.

The special positions of the limbs advocated by Mr. Delorme for the relaxation of the nerve were strongly criticized at first, but they have gained much favor now. A few weeks after the nerve suture the limb is quite easily made to regain its normal and straight direction.

Practically, also, the removal of all the injured part of the nerve, and the bringing together of sound regions only, proves an excellent thing, in spite of the corresponding loss of substance. In fact this technic gives 70 and even 80 per cent. of excellent results, whereas the incomplete excisions of the neuromae and gliomae of the injured extremities are responsible for all the bad results obtained in nerve sutures during 1915-1916.

Finally the difficulty in distinguishing a sound part of a nerve from an injured one has been much exaggerated. Since 1916, many neurologists have ascertained that the examination " de visu " proves the simplest and surest method of determining the limits of the necessary excision.

The freeing of a certain length of nerve and its relaxation, thanks to favorable positions of the limbs, have allowed nerve suture in many cases where formerly grafting would have been deemed necessary; and nerve grafts are not to be compared, as to their results, to simple and direct approximation of the extremities.

Lateral neuromae following lateral excoriation, or central neuromae consecutive to some bruising of the nerve, must be removed totally before suturing, since they interfere with the repairing process of the nerve.

Mr. WALTHER (*from Paris*) has treated surgically many nerve injuries since 1914. He deplores that so many wounded men remain crippled because they suffer from some non-operated nerve lesions. Too many surgeons still fear to resort to the technic of resection and suture, or graft, even in cases where the medical treatment has brought no improvement.

Mr. Walther considers, as does Mr. Delorme, that both ends of the injured nerve must be carefully resected, and that only sound regions must be approximated. In cases of lateral or central neuromae these must be resected. But surgical liberation seems advisable and effective not only in cases of simple constriction, but also in bruising or semi-crushing of the nerve. In such cases, the resection is perhaps somewhat too " radical ", except when such lesions are accompanied by violent pain along the track of the nerve.

Mr. Walther finds it most effective to bathe the nerve with warm normal saline during the whole operation. He insists upon the necessity of separating the nerve most carefully, after the suturing, from all surrounding soft parts. Muscular sheaths prove most convenient for this if perfectly wholesome; if this requirement cannot be fulfilled, rubber sheaths should be resorted to.

Mr. L. Sencert (*from Nancy*) reports the results obtained in using heterografts in the treatment of injured nerves. The restoration of a sectioned nerve should be tried as soon as possible, by approximating the extremities by means of a loose neurolemma suture. The fascia should be disposed near each other, but not pressed tightly against each other, so as to allow of the development of the repairing neuroglioma.

If surgical treatment is not resorted to immediately, a dense fibrous tissue, completely deprived of any " neurite ", develops between the sectioned ends, and this must be resected until high above the neuroma.

Mr. Sencert, together with Mr. Nageotte, considers that grafts must necessarily be resorted to, to make up for the resected substance. The authors have experimented with dead heterografts, as long as 12 and 13 cm. in some cases. The results seem excellent, but they will be truly convincing only when more time shall have elapsed since these interventions occurred.

Mr. Forgue (*from Montpellier*) remarks how interesting and difficult it proves to be informed of the remote results of the interventions performed on injured nerves, — the sensory and motor, together with the functional restoration, improve so slowly and need such a long time to be completed.

22 remote results have been obtained. Out of 130 cases operated on by Mr. Forgue, 3 show excellent motor restoration; 10 show a partial sensory restoration — these were operated from 5 to 14 months previously; 9 cases show as yet no signs of restoration at all.

Mr. Dujarier (*from Paris*) has performed 30 homoplastic nerve grafts, the results of which are mostly unknown to him; but several returns of normal electrical contractility, and at least one return of motion, after grafting the radial nerve, have been observed.

Mr. Gernez (*from Paris*) has performed 120 interventions upon injured nerves : 45 liberations, 71 sutures, 2 removals of intratruncular projectiles, 2 sympathicectomies and 1 injection of alcohol in a case of causalgia.

His technic does not differ from that generally used by most surgeons, but he resorts frequently to sheaths of fatty tissue after the liberation and suture of the nerves.

Mr. Mauclaire (*from Paris*) describes two modes of nerve autografts : autograft with a graft taken from the sciatic, and bridged autograft.

In the first case a fragment was removed from the trunk of the sciatic nerve from the medium part of the thigh. This fragment consisted of about one-third of the trunk; it was grafted between two ends of the external popliteal sciatic which proved too far apart from each other to be approximated directly.

In the case of " bridged autograft ", Mr. Mauclaire propped the injured nerve by means of the trunk of some adjacent nerve. For instance, he spanned some space in the radial either with the musculo-cutaneous or the internal brachial-cutaneous. This coupling of two nerves favors, perhaps, the regeneration of the injured nerve by a sort of neurotropic power.

Mr. Mauclaire highly recommends the " topographic suture ". Many neurologists consider that quite a series of " topographic " circular zones exists in a nerve, corresponding to each of the muscles innervated by the nerve. While suturing two ends of a nerve, great care must be taken not to twist these extremities so that the zones of the central end correspond exactly to those of the distal one.

When the nerve is surrounded by much fibrous tissue, resulting from both perinervous and intranervous hematomae, Mr. Mauclaire resorts to a large peripheral liberation, and sheathes the trunk with a fenestrated rubber sheet. The first case treated in that way in 1915 was followed by very good results.

Mr. Mauclaire describes next the technic of the tendinous anastomoses, in cases of definitive radial paralysis.

Tendinous anastomoses may be resorted to in cases of disablement of the wrist following some paralysis of the radial nerve.

Mr. Mauclaire liberates the tendons of the large and small palmar, and of the anterior cubital muscles on the palmar aspect of the wrist, as low down as possible. These tendons are next passed under the skin and sutured to the tendons of the radial muscles and extensors on the dorsal aspect.

For a fortnight the hand is fixed on a splint in hyperextension, the fingers being mobilized every day to avoid stiffness in the joints.

Mr. E. VILLARD (*from Lyon*) studies the suture and regeneration of the nerve in cases of complete section of the radial.

Mr. Villard operated on 20 such cases, but only 8 of these could be reexamined by him. In 4 of them he observed the normal return of motion. The sutures had taken place respectively 6 days, 43 days, 4 and 6 months after receipt of the wound. The first voluntary movements appeared from 8 to 11 months after the intervention.

The return of motion always progressed most regularly from the central to the distal ends, and according to the anatomical disposition of the nerve; the muscles innervated by the shorter branches were the first to regain voluntary contractility. The return of function was more rapid in cases of early suture.

In 2 cases, the suture was performed before the cicatrization of an infected focus was complete, and normal regeneration of the nerve followed in both cases.

Mr. Wiart (*from Paris*) has been informed of the results of 86 interventions following war wounds of the radial nerve. Among these are 25 sutures and 61 surgical liberations.

*Sutures.*

| | | | |
|---|---|---|---|
| Healings | 5 | or | 20 o/o |
| Marked improvement of the motor functions | 5 | " | 20 o/o |
| Improvement in the return of sensibility or electrical contractility | 2 | " | 8 o/o |
| Failures | 13 | " | 52 o/o |

The lesions were all old when they were operated upon by the author. The wounds and fractures had generally suppurated for a long time. Four men among the 5 who healed completely were operated from 150 to 330 days after receipt of the wound.

In 4 cases the section of the nerve was incomplete; among these one healing occurred. All other cases had complete sections.

The first movements appeared twice after 3 months, once after 10 months, and once after 17 months and a half.

The recovery was complete after 10, 15, 21, 22 and 24 months according to the cases.

*Surgical Liberations.* The results were as follows:

| | | | |
|---|---|---|---|
| Healings | 20 | or | 33.70 o/o |
| Marked improvement in the return of motion | 15 | " | 24.6 o/o |
| Improvement in the electrical contractility | 11 | " | 18.09 o/o |
| Failures | 15 | " | 24.59 o/o |

Five cases operated upon less than 3 months after receipt of the wound healed.

Among the failures, all the lesions were more than five months old.

The earlier the lesions are operated upon the sooner do the first voluntary movements appear, and the sooner also is recovery complete.

M. Vitrac (*from Bordeaux*) had resorted to nerve sutures as long ago as 1915, but induced by the reports of the physiologists and neuro-pathologists of the "Ecole de Bordeaux", intentionally gave up this sort of treatment during 1916 and 1917.

He has, however, had the opportunity of examining more than 100 long-standing cases of nerve lesions treated in different hospitals. All those who had not undergone nerve suture did not show any improvement at all, while the condition of four men, who had undergone suture, was markedly improving.

M. Salva Mercadé (*from Paris*) has treated 37 war lesions of nerves and has been able to observe 17 remote results of interventions.

As to the treatment, he insists upon the necessity of insulating carefully the operated nerve either by coating it with a fatty, pediculated sheath, removed from the sub-cutaneous tissue, or by placing it on a muscular or aponeurotic layer.

In one case, operated upon as late as 6 months after receipt of the wound, complete recovery was obtained.

The post-operative electrical treatment must be carried on for a very long time. In a case of lesion of the radial nerve, the benefits of this treatment appeared only after 18 months' time. Then, however, it produced such rapid improvement that the man was able to take up active service two months afterward.

The 17 remote results reported by the author include 7 sutures, 3 removals of neuromae, and 7 surgical liberations.

The nerves concerned in the sutures were the radial, the ulnar, the median, the internal cutaneous brachial and the external popliteal sciatic. 3 healings were obtained and 1 marked improvement.

The removals of neuromae were practised four months after receipt of the wound and resulted in 1 healing, and 1 improvement.

The results of the 7 liberations were as follows: 4 complete recoveries, 2 improvements, and in one case no change at all.

